# Supplementary material for: The weakness of fragility index exposed in an analysis of the traumatic brain injury management guidelines: A meta-epidemiological and simulation study
Source: PLoS One. 2020 Aug 18;15(8):e0237879. doi: 10.1371/journal.pone.0237879 (PMC7433866; doi:10.1371/journal.pone.0237879)
Supplement: S1 File — (DOCX) [file pone.0237879.s001.docx]

**S1 Table. Included trials and study outcomes**

| **Topic** | **Study ID** | **First author** | **Year** | **Outcome** |
| --- | --- | --- | --- | --- |
| **Decompressive craniectomy** | 1 | Cooper^1^ | 2011 | EGS at 6 months |
|  | 2 | Jiang^2^ | 2005 | GOS at 6 months |
|  | 3 | Qiu^3^ | 2009 | GOS at 6 months |
|  | 4 | Hutchinson^4^ | 2016 | EGS at 6 months |
| **Prophylactic hypothermia** | 5 | Aibiki^5^ | 2000 | GOS at 6 months |
|  | 6 | Clifton^6^ | 1993 | GOS at 3 months |
|  | 7 | Clifton^7^ | 2001 | GOS at 6 months |
|  | 8 | Clifton^8^ | 2011 | GOS at 6 months |
|  | 9 | Jiang^9^ | 2000 | GOS at 1 year |
|  | 10 | Marion^10^ | 1997 | GOS at 1 year |
|  | 11 | Qiu^11^ | 2005 | GOS at 2 year |
|  | 12 | Qiu^12^ | 2007 | Neurological outcome at 1 year |
|  | 13 | Qiu^13^ | 2006 | GOS at 6 months |
|  | 14 | Smrcka^14^ | 2005 | GOS at 6 months |
|  | 15 | Zhao^15^ | 2011 | GOS at 3 months |
|  | 16 | Jiang^16^ | 2006 | GOS at 6 months |
|  | 17 | Harris^17^ | 2009 | Mortality at 28 days |
| **Hyperosmolar therapy** | 18 | Cottenceau^18^ | 2011 | GOS at 6 months |
|  | 19 | Schwartz^19^ | 1984 | Survival at 1 year |
| **Anesthetics, analgesics and sedatives** | 20 | Ward^20^ | 1985 | GOS at 1 year |
|  | 21 | Kelly^21^ | 1999 | GOS at 6 months |
|  | 22 | Ghori^22^ | 2007 | GOS at 3 months |
| **Steroids** | 23 | Roberts^23^ | 2004 | Mortality at 2 weeks |
|  | 24 | Edwards^24^ | 2005 | GOS at 6 months |
|  | 25 | Marshall^25^ | 1998 | GOS at 6 months |
|  | 26 | Saul^26^ | 1981 | Outcomes at 6 months (similar to GOS) |
|  | 27 | Gaab^27^ | 1994 | Modified GOS at 10-14 months |
| **Nutrition** | 28 | Rapp^28^ | 1983 | Neurological state at 1 year (similar to GOS) |
|  | 29 | Taylor^29^ | 1999 | GOS at 6 months |
|  | 30 | Acosta-Escribano^30^ | 2010 | Mortality at 30 days |
|  | 31 | Bilotta^31^ | 2008 | GOS at 6 months |
|  | 32 | Coester^32^ | 2010 | GOS at 6 months |
|  | 33 | Yang^33^ | 2009 | GOS at 6 months |
|  | 34 | Young^34^ | 1996 | GCS at 28 days |
|  | 35 | Grahm^35^ | 1989 | Incidence of bacterial infections |
|  | 36 | Hadley^36^ | 1986 | Follow up GCS |
|  | 37 | Young^37^ | 1987 | GOS at 1 year |

| **Infection prophylaxis** | 38 | Bouderka^38^ | 2004 | Pneumonia/Mortality |
| --- | --- | --- | --- | --- |
|  | 39 | Seguin^39^ | 2014 | Pneumonia/Mortality |
|  | 40 | Sirvent^40^ | 1997 | Pneumonia/Mortality |
| **Seizure prophylaxis** | 41 | Temkin^41^ | 1990 | Patients with Seizure |
|  | 42 | Dikmen^42^ | 1991 | Patients with Seizure |
|  | 43 | Young^43^ | 1983 | Patients with Seizure |

EGS, Extended Glasgow Outcome Score; GOS, Glasgow Outcome Score; GCS, Glasgow Coma Scale

**S2 Table. Fragility analysis of included trials**

| **Topic** | **Study ID** | **Size** | **N(Int)** | **N(Comp)** | **E(Int)** | **E(Comp)** | **RR** | **N(LFU)** | **P(Fisher)** | **FI_F_** | **P(RR)** | **FI_RR_** |
| --- | --- | --- | --- | --- | --- | --- | --- | --- | --- | --- | --- | --- |
| **Decompressive craniectomy** | 1 | 155 | 73 | 82 | 51 | 42 | 0.73 | 0 | 0.022 | 3 | 0.0095 | 5 |
|  | 2 | 486 | 245 | 241 | 96 | 70 | 0.74 | 0 | 0.022 | 4 | 0.0098 | 8 |
|  | 3 | 74 | 37 | 37 | 21 | 12 | 0.57 | 0 | 0.061 | -1 | 0.022 | 2 |
|  | 4 | 398 | 202 | 196 | 86 | 65 | 0.78 | 9 | 0.063 | -1 | 0.028 | 3 |
| **Prophylactic hypothermia** | 5 | 26 | 15 | 11 | 12 | 4 | 0.45 | 0 | 0.043 | 1 | 0.03 | 1 |
|  | 6 | 46 | 24 | 22 | 12 | 8 | 0.73 | 1 | 0.39 | -4 | 0.18 | -3 |
|  | 7 | 392 | 199 | 193 | 108 | 102 | 0.97 | 24 | 0.84 | -17 | 0.39 | -14 |
|  | 8 | 97 | 52 | 45 | 31 | 25 | 0.93 | 0 | 0.84 | -8 | 0.34 | -6 |
|  | 9 | 87 | 43 | 44 | 20 | 12 | 0.59 | 0 | 0.077 | -1 | 0.035 | 1 |
|  | 10 | 80 | 40 | 42 | 24 | 16 | 0.63 | 1 | 0.076 | -1 | 0.027 | 2 |
|  | 11 | 86 | 43 | 43 | 28 | 16 | 0.57 | - | 0.017 | 2 | 0.0069 | 5 |
|  | 12 | 80 | 40 | 40 | 28 | 19 | 0.68 | - | 0.069 | -1 | 0.024 | 2 |
|  | 13 | 90 | 45 | 45 | 33 | 23 | 0.70 | 0 | 0.05 | 1 | 0.018 | 3 |
|  | 14 | 72 | 37 | 35 | 30 | 18 | 0.63 | 0 | 0.012 | 3 | 0.0063 | 5 |
|  | 15 | 81 | 40 | 41 | 30 | 21 | 0.68 | 0 | 0.038 | 1 | 0.016 | 3 |
|  | 16 | 215 | 108 | 107 | 47 | 31 | 0.67 | 0 | 0.033 | 2 | 0.015 | 4 |
|  | 17 | 25 | 12 | 13 | 6 | 4 | 0.62 | 0 | 0.43 | -3 | 0.17 | -2 |
| **Hyperosmolar therapy** | 18 | 47 | 22 | 25 | 5 | 11 | 0.52 | 0 | 0.22 | -2 | 0.072 | -1 |
|  | 19 | 59 | 28 | 31 | 16 | 15 | 0.85 | 0 | 0.6 | -6 | 0.25 | -5 |
| **Anesthetics, analgesics and sedatives** | 20 | 53 | 27 | 26 | 11 | 10 | 0.94 | 0 | 1 | -7 | 0.43 | -6 |
|  | 21 | 42 | 23 | 19 | 12 | 9 | 0.91 | 8 | 1 | -6 | 0.38 | -5 |
|  | 22 | 28 | 15 | 13 | 8 | 7 | 1.01 | 0 | 1 | -6 | 0.49 | -5 |
| **Steroids** | 23 | 10008 | 5007 | 5001 | 1052 | 893 | 0.85 | 44 | 7.3e-5 | 79 | 3.5e-05 | 92 |
|  | 24 | 10008 | 5007 | 5001 | 1828 | 1728 | 0.95 | 454 | 0.043 | 4 | 0.021 | 19 |
|  | 25 | 957 | 482 | 475 | 220 | 205 | 0.95 | 39 | 0.47 | -19 | 0.22 | -14 |
|  | 26 | 100 | 50 | 50 | 31 | 26 | 0.84 | 0 | 0.42 | -6 | 0.16 | -4 |
|  | 27 | 300 | 133 | 136 | 19 | 21 | 0.93 | 31 | 0.86 | -10 | 0.4 | -8 |
| **Nutrition** | 28 | 38 | 20 | 18 | 12 | 7 | 0.65 | 1 | 0.33 | -3 | 0.11 | -2 |
|  | 29 | 82 | 41 | 41 | 25 | 28 | 0.89 | 0 | 0.64 | -7 | 0.24 | -5 |
|  | 30 | 104 | 47 | 54 | 6 | 9 | 0.77 | 0 | 0.78 | -5 | 0.29 | -4 |
|  | 31 | 97 | 48 | 49 | 23 | 21 | 0.89 | 0 | 0.69 | -8 | 0.31 | -6 |
|  | 32 | 88 | 42 | 46 | 16 | 13 | 0.74 | 9 | 0.37 | -5 | 0.16 | -4 |
|  | 33 | 240 | 121 | 119 | 34 | 26 | 0.78 | 7 | 0.3 | -6 | 0.13 | -4 |
|  | 34 | 68 | 33 | 35 | 4 | 9 | 0.47 | 0 | 0.22 | -2 | 0.086 | -1 |
|  | 35 | 32 | 17 | 15 | 3 | 14 | 0.19 | - | 1.9e-5 | 8 | 8.1e-4 | 9 |
|  | 36 | 45 | 24 | 21 | 17 | 15 | 1.01 | - | 1 | -7 | 0.48 | -6 |
|  | 37 | 51 | 28 | 23 | 9 | 10 | 0.74 | 0 | 0.56 | -5 | 0.2 | -4 |
| **Infection prophylaxis** | 38 | 62 | 31 | 31 | 18 | 19 | 0.95 | 0 | 1 | -8 | 0.4 | -6 |
|  | 39 | 167 | 85 | 82 | 24 | 20 | 0.86 | 17 | 0.6 | -8 | 0.29 | -6 |
|  | 40 | 100 | 50 | 50 | 12 | 25 | 0.48 | 0 | 0.012 | 3 | 0.0055 | 5 |
| **Seizure prophylaxis** | 41 | 404 | 208 | 196 | 7 | 26 | 0.25 | 0 | 3.9e-4 | 9 | 4.6e-4 | 10 |
|  | 42 | 404 | 208 | 196 | 3 | 23 | 0.12 | 199 | 1.9e-5 | 10 | 2.7e-4 | 12 |
|  | 43 | 214 | 119 | 95 | 13 | 8 | 0.77 | 11 | 0.65 | -5 | 0.27 | -4 |

Int, intervention; Comp, comparator; N(*), group size; E(*), number of events; LFU, lost to follow-up; FI, fragility index; RR, relative risk

**REFERENCES**

1. Cooper DJ, Rosenfeld JV. Does decompressive craniectomy improve outcomes in patients with diffuse traumatic brain injury? *Med J Aust* 2011;194(9):437-8.

2. Jiang JY, Xu W, Li WP, et al. Efficacy of standard trauma craniectomy for refractory intracranial hypertension with severe traumatic brain injury: a multicenter, prospective, randomized controlled study. *J Neurotrauma* 2005;22(6):623-8. doi: 10.1089/neu.2005.22.623

3. Qiu W, Guo C, Shen H, et al. Effects of unilateral decompressive craniectomy on patients with unilateral acute post-traumatic brain swelling after severe traumatic brain injury. *Crit Care* 2009;13(6):R185. doi: 10.1186/cc8178 [published Online First: 2009/11/23]

4. Hutchinson PJ, Kolias AG, Timofeev IS, et al. Trial of Decompressive Craniectomy for Traumatic Intracranial Hypertension. *N Engl J Med* 2016;375(12):1119-30. doi: 10.1056/NEJMoa1605215 [published Online First: 2016/09/07]

5. Aibiki M, Maekawa S, Yokono S. Moderate hypothermia improves imbalances of thromboxane A2 and prostaglandin I2 production after traumatic brain injury in humans. *Crit Care Med* 2000;28(12):3902-6. doi: 10.1097/00003246-200012000-00029

6. Clifton GL, Allen S, Barrodale P, et al. A phase II study of moderate hypothermia in severe brain injury. *J Neurotrauma* 1993;10(3):263-71; discussion 73. doi: 10.1089/neu.1993.10.263

7. Clifton GL, Miller ER, Choi SC, et al. Lack of effect of induction of hypothermia after acute brain injury. *N Engl J Med* 2001;344(8):556-63. doi: 10.1056/nejm200102223440803

8. Clifton GL, Valadka A, Zygun D, et al. Very early hypothermia induction in patients with severe brain injury (the National Acute Brain Injury Study: Hypothermia II): a randomised trial. *Lancet Neurol* 2011;10(2):131-9. doi: 10.1016/s1474-4422(10)70300-8 [published Online First: 2010/12/17]

9. Jiang J, Yu M, Zhu C. Effect of long-term mild hypothermia therapy in patients with severe traumatic brain injury: 1-year follow-up review of 87 cases. *J Neurosurg* 2000;93(4):546-9. doi: 10.3171/jns.2000.93.4.0546

10. Marion DW, Penrod LE, Kelsey SF, et al. Treatment of traumatic brain injury with moderate hypothermia. *N Engl J Med* 1997;336(8):540-6. doi: 10.1056/nejm199702203360803

11. Qiu WS, Liu WG, Shen H, et al. Therapeutic effect of mild hypothermia on severe traumatic head injury. *Chin J Traumatol* 2005;8(1):27-32.

12. Qiu W, Zhang Y, Sheng H, et al. Effects of therapeutic mild hypothermia on patients with severe traumatic brain injury after craniotomy. *J Crit Care* 2007;22(3):229-35. doi: 10.1016/j.jcrc.2006.06.011 [published Online First: 2007/01/31]

13. Qiu W, Shen H, Zhang Y, et al. Noninvasive selective brain cooling by head and neck cooling is protective in severe traumatic brain injury. *J Clin Neurosci* 2006;13(10):995-1000. doi: 10.1016/j.jocn.2006.02.027

14. Smrcka M, Vidlák M, Máca K, et al. The influence of mild hypothermia on ICP, CPP and outcome in patients with primary and secondary brain injury. *Acta Neurochir Suppl* 2005;95:273-5. doi: 10.1007/3-211-32318-x_56

15. Zhao QJ, Zhang XG, Wang LX. Mild hypothermia therapy reduces blood glucose and lactate and improves neurologic outcomes in patients with severe traumatic brain injury. *J Crit Care* 2011;26(3):311-5. doi: 10.1016/j.jcrc.2010.08.014 [published Online First: 2010/10/02]

16. Jiang JY, Xu W, Li WP, et al. Effect of long-term mild hypothermia or short-term mild hypothermia on outcome of patients with severe traumatic brain injury. *J Cereb Blood Flow Metab* 2006;26(6):771-6. doi: 10.1038/sj.jcbfm.9600253

17. Harris OA, Muh CR, Surles MC, et al. Discrete cerebral hypothermia in the management of traumatic brain injury: a randomized controlled trial. *J Neurosurg* 2009;110(6):1256-64. doi: 10.3171/2009.1.Jns081320

18. Cottenceau V, Masson F, Mahamid E, et al. Comparison of effects of equiosmolar doses of mannitol and hypertonic saline on cerebral blood flow and metabolism in traumatic brain injury. *J Neurotrauma* 2011;28(10):2003-12. doi: 10.1089/neu.2011.1929 [published Online First: 2011/09/23]

19. Schwartz ML, Tator CH, Rowed DW, et al. The University of Toronto head injury treatment study: a prospective, randomized comparison of pentobarbital and mannitol. *Can J Neurol Sci* 1984;11(4):434-40. doi: 10.1017/s0317167100045960

20. Ward JD, Becker DP, Miller JD, et al. Failure of prophylactic barbiturate coma in the treatment of severe head injury. *J Neurosurg* 1985;62(3):383-8. doi: 10.3171/jns.1985.62.3.0383

21. Kelly DF, Goodale DB, Williams J, et al. Propofol in the treatment of moderate and severe head injury: a randomized, prospective double-blinded pilot trial. *J Neurosurg* 1999;90(6):1042-52. doi: 10.3171/jns.1999.90.6.1042

22. Ghori KA, Harmon DC, Elashaal A, et al. Effect of midazolam versus propofol sedation on markers of neurological injury and outcome after isolated severe head injury: a pilot study. *Crit Care Resusc* 2007;9(2):166-71.

23. Roberts I, Yates D, Sandercock P, et al. Effect of intravenous corticosteroids on death within 14 days in 10008 adults with clinically significant head injury (MRC CRASH trial): randomised placebo-controlled trial. *Lancet* 2004;364(9442):1321-8. doi: 10.1016/s0140-6736(04)17188-2

24. Edwards P, Arango M, Balica L, et al. Final results of MRC CRASH, a randomised placebo-controlled trial of intravenous corticosteroid in adults with head injury-outcomes at 6 months. *Lancet* 2005;365(9475):1957-9. doi: 10.1016/s0140-6736(05)66552-x

25. Marshall LF, Maas AI, Marshall SB, et al. A multicenter trial on the efficacy of using tirilazad mesylate in cases of head injury. *J Neurosurg* 1998;89(4):519-25. doi: 10.3171/jns.1998.89.4.0519

26. Saul TG, Ducker TB, Salcman M, et al. Steroids in severe head injury: A prospective randomized clinical trial. *J Neurosurg* 1981;54(5):596-600. doi: 10.3171/jns.1981.54.5.0596

27. Gaab MR, Trost HA, Alcantara A, et al. "Ultrahigh" dexamethasone in acute brain injury. Results from a prospective randomized double-blind multicenter trial (GUDHIS). German Ultrahigh Dexamethasone Head Injury Study Group. *Zentralbl Neurochir* 1994;55(3):135-43.

28. Rapp RP, Young B, Twyman D, et al. The favorable effect of early parenteral feeding on survival in head-injured patients. *J Neurosurg* 1983;58(6):906-12. doi: 10.3171/jns.1983.58.6.0906

29. Taylor SJ, Fettes SB, Jewkes C, et al. Prospective, randomized, controlled trial to determine the effect of early enhanced enteral nutrition on clinical outcome in mechanically ventilated patients suffering head injury. *Crit Care Med* 1999;27(11):2525-31. doi: 10.1097/00003246-199911000-00033

30. Acosta-Escribano J, Fernández-Vivas M, Grau Carmona T, et al. Gastric versus transpyloric feeding in severe traumatic brain injury: a prospective, randomized trial. *Intensive Care Med* 2010;36(9):1532-9. doi: 10.1007/s00134-010-1908-3 [published Online First: 2010/05/22]

31. Bilotta F, Caramia R, Cernak I, et al. Intensive insulin therapy after severe traumatic brain injury: a randomized clinical trial. *Neurocrit Care* 2008;9(2):159-66. doi: 10.1007/s12028-008-9084-9

32. Coester A, Neumann CR, Schmidt MI. Intensive insulin therapy in severe traumatic brain injury: a randomized trial. *J Trauma* 2010;68(4):904-11. doi: 10.1097/TA.0b013e3181c9afc2

33. Yang M, Guo Q, Zhang X, et al. Intensive insulin therapy on infection rate, days in NICU, in-hospital mortality and neurological outcome in severe traumatic brain injury patients: a randomized controlled trial. *Int J Nurs Stud* 2009;46(6):753-8. doi: 10.1016/j.ijnurstu.2009.01.004 [published Online First: 2009/02/20]

34. Young B, Ott L, Kasarskis E, et al. Zinc supplementation is associated with improved neurologic recovery rate and visceral protein levels of patients with severe closed head injury. *J Neurotrauma* 1996;13(1):25-34. doi: 10.1089/neu.1996.13.25

35. Grahm TW, Zadrozny DB, Harrington T. The benefits of early jejunal hyperalimentation in the head-injured patient. *Neurosurgery* 1989;25(5):729-35. doi: 10.1097/00006123-198911000-00007

36. Hadley MN, Grahm TW, Harrington T, et al. Nutritional support and neurotrauma: a critical review of early nutrition in forty-five acute head injury patients. *Neurosurgery* 1986;19(3):367-73. doi: 10.1227/00006123-198609000-00006

37. Young B, Ott L, Haack D, et al. Effect of total parenteral nutrition upon intracranial pressure in severe head injury. *J Neurosurg* 1987;67(1):76-80. doi: 10.3171/jns.1987.67.1.0076

38. Bouderka MA, Fakhir B, Bouaggad A, et al. Early tracheostomy versus prolonged endotracheal intubation in severe head injury. *J Trauma* 2004;57(2):251-4. doi: 10.1097/01.ta.0000087646.68382.9a

39. Seguin P, Laviolle B, Dahyot-Fizelier C, et al. Effect of oropharyngeal povidone-iodine preventive oral care on ventilator-associated pneumonia in severely brain-injured or cerebral hemorrhage patients: a multicenter, randomized controlled trial. *Crit Care Med* 2014;42(1):1-8. doi: 10.1097/CCM.0b013e3182a2770f

40. Sirvent JM, Torres A, El-Ebiary M, et al. Protective effect of intravenously administered cefuroxime against nosocomial pneumonia in patients with structural coma. *Am J Respir Crit Care Med* 1997;155(5):1729-34. doi: 10.1164/ajrccm.155.5.9154884

41. Temkin NR, Dikmen SS, Wilensky AJ, et al. A randomized, double-blind study of phenytoin for the prevention of post-traumatic seizures. *N Engl J Med* 1990;323(8):497-502. doi: 10.1056/nejm199008233230801

42. Dikmen SS, Temkin NR, Miller B, et al. Neurobehavioral effects of phenytoin prophylaxis of posttraumatic seizures. *JAMA* 1991;265(10):1271-7.

43. Young B, Rapp RP, Norton JA, et al. Failure of prophylactically administered phenytoin to prevent post-traumatic seizures in children. *Childs Brain* 1983;10(3):185-92. doi: 10.1159/000120113
